# Update on the current status of onchocerciasis in Côte d’Ivoire following 40 years of intervention: Progress and challenges

**DOI:** 10.1371/journal.pntd.0006897

**Published:** 2018-10-23

**Authors:** Benjamin G. Koudou, Marie-Madeleine Kouakou, Allassane F. Ouattara, Souleymane Yeo, Pierre Brika, Aboulaye Meite, Elvis Aba, Christopher L. King, Roger Kouakou, Gary J. Weil, Peter U. Fischer

**Affiliations:** 1 Laboratory of Biology and Animal Cytology, Training and Research Unit in Natural Sciences, Nangui Abrogoua University, Abidjan, Côte d’Ivoire; 2 Centre Suisse de Recherches Scientifiques en Côte d’Ivoire, Research and Development Department, Abidjan, Côte d’Ivoire; 3 Vector Biology Department, Liverpool School of Tropical Medicine, Pembroke Place, United Kingdom; 4 National Onchocerciasis Control Programme, Ministry of Health and Public hygiene in Côte d’Ivoire, Abidjan, Côte d’Ivoire; 5 Case Western Reserve University, Center for Global Health and Disease, Cleveland, Ohio, United States of America; 6 Washington University School of Medicine, Department of Medicine, Infectious Diseases Division, St. Louis, Missouri, United States of America; Negrar Hospital, ITALY

## Abstract

**Background:**

Onchocerciasis control in Côte d’Ivoire started with aerial insecticide spraying in 1974 and continued with community directed treatment with ivermectin (CDTi) from 1992 to the present. Onchocerciasis and lymphatic filariasis (LF) are co-endemic in 46 of the 81 health districts in the country. Fourteen and 12 districts are endemic for only LF or onchocerciasis, respectively. This paper aims to review the impact of past interventions on onchocerciasis in Côte d’Ivoire between 1975 and 2013, and review plans for disease elimination.

**Methods:**

We reviewed microfilaria (MF, skin snip) prevalence and community microfilarial load (CMFL) data from published reports from 53 health districts during two major epidemiological assessment periods. Data from 1975 through 1991 provided information on the impact of vector control, and data from 1992 through 2016 provided information on the impact of CDTi.

**Results:**

Weekly aerial insecticide spraying in 8 endemic districts between 1975 and 1991 reduced the overall MF prevalence by 68.1% from 43.5% to 13.9%. The CMFL also decreased in 7 out of 8 surveyed communities by 95.2% from 9.24 MF/snip to 0.44 MF/snip. Ivermectin distribution started in 1992. The coverage targets for control (65% of the total population) was reached in most endemic districts, and some areas achieved 80% coverage. Two sets of surveys were conducted to assess the impact of CDTi. Results from the first repeat surveys showed a significant decrease in overall MF prevalence (by 75.7%, from 41.6% to 10.1%). The second follow-up evaluation showed further improvement in most endemic districts and also documented major reductions in CMFL compared to baseline.

**Conclusions:**

Extensive data collected over many years document the very significant impact of interventions conducted by the National Onchocerciasis and other Eyes Diseases Control Programme during challenging times with periods of civil unrest. The Health Ministry has now integrated efforts to control neglected tropical diseases and adopted the goal of onchocerciasis elimination.

## Author summary

Onchocerciasis has recently been targeted for elimination in Côte d’Ivoire. With support of international donors the country’s integrated Neglected Tropical Diseases (NTD) program has recently achieved 100% geographical coverage for mass drug administration in districts endemic for lymphatic filariasis and onchocerciasis. From 1975 to 1991 onchocerciasis control in Côte d’Ivoire was based on weekly aerial spraying of insecticide on targeted rivers. From 1992 to present, community directed treatment with ivermectin by community drug distributors was implemented in most endemic areas. Although the country experienced periods of civil disturbance that interrupted distribution of ivermectin for several years, significant progress was made during this time toward onchocerciasis elimination. There have been significant reductions in both microfilarial prevalences and community microfilarial loads in sentinel villages that had implementation of vector control and/or mass drug administration coverage rates of at least 65%. Although onchocerciasis persists in some areas, changes in management of the programme in 2015 together with improved political stability and increased support from key donors may lead to its elimination over the next 10 years.

## Introduction

Onchocerciasis (also known as “river blindness”) is a serious public health problem that has hindered socioeconomic development in endemic areas in sub-Saharan Africa [1]. The disease is caused by the filarial parasite *Onchocerca volvulus* that is transmitted by *Simulium* black flies. This infection causes excess mortality [2] and significant morbidity that can include severe dermatitis with itching, skin depigmentation, subcutaneous nodules where adult parasites are located, and visual impairment [3].

The initial efforts of the Onchocerciasis Control Programme in West Africa (OCP) focused on vector control through aerial application of larvicides in black fly breeding sites from 1974 to 1992 in 11 West African countries [4]. Vector control had a positive impact on controlling onchocerciasis in many savannah areas, but it was difficult to implement in densely forested areas. Since 1987 the recommended control strategy for onchocerciasis in Africa has been annual or semi-annual mass treatment with ivermectin, which kills the microfilarial progeny that cause skin disease and blindness. From 2002, when OCP was taken over by the African Programme for Onchocerciasis Control (APOC) through 2015 when APOC closed, more than 1 billion ivermectin treatments were administered, and more than 2 million cases of blindness were prevented [5]. Successes against onchocerciasis in sub-Saharan Africa and Latin America can be mainly attributed to the extensive and sustained mass treatment programs [5]. Since 2016, efforts to control and eliminate onchocerciasis in Africa have been managed by the Expanded Special Project for Elimination of Neglected Tropical Diseases (ESPEN).

Côte d’Ivoire is highly endemic for a number of Neglected Tropical Diseases (NTD) including onchocerciasis and lymphatic filariasis (LF). The two diseases are co-endemic in 46 of the country’s 81 health districts. Fourteen and 12 districts are endemic for only LF or onchocerciasis, respectively, and 9 districts are not endemic for either disease. The total population of the country was in 2015 25,236,292, and the estimated population living within 5 km from the rivers including villages with less than 2,000 inhabitants that are considered to be at especially high risk for onchocerciasis was 2,867,122. Côte d’Ivoire experienced several periods of civil disturbance in recent years that peaked in 2002 and in 2011. This has had negative impacts on the economy and on the public health infrastructure [6], and many international research and aid organisations left the country during this time. Political stability has improved, but it could take a decade or more to rebuild the public health and research infrastructures.

Control of onchocerciasis started with the beginning of the OCP in 1974, but it was initially limited to the northern (savannah) region of the country. Vector control activities reached the central region from 1986 to 1991 [6]. In 1992, most countries supported by OCP including Côte d’Ivoire started mass distribution of ivermectin through community directed treatment (CDTi) with a single annual oral dose of 150 μg/kg body weight. The closure of OCP in 2002 coincided with the start of the civil war in Côte d’Ivoire. This made it impossible for the National Onchocerciasis Control Programme to sustain onchocerciasis control activities between 2002 and 2007, especially in endemic districts of the northern, central and western regions that were occupied by rebel forces. This was a serious problem because onchocerciasis was the second most common cause of blindness in the country after cataract [8]. Fortunately, support from APOC together with a calmer political environment helped to restart CDTi in the troubled areas in 2008.

Côte d’Ivoire is now taking steps to eliminate onchocerciasis. The country’s integrated NTD program has recently achieved 100% geographical coverage for mass drug administration (MDA) in all districts endemic for LF and onchocerciasis (only 8 districts are endemic for onchocerciasis only). The purpose of this paper is to review published data on the impact of vector control and CDTi activities that started in endemic districts in 1975 and in 1992, respectively, and to summarize the most recent data on the distribution and prevalence of onchocerciasis in the country. We conducted this exercise to support the onchocerciasis eliminate program by identifying key gaps in the data and by highlighting potential problem areas that may require special attention.

## Materials and methods

### Study areas

The country is divided into four geographical zones: forest, pre-forest, savannah and mountain that each have specific ecological features and climates. Historically, blinding onchocerciasis occurred mainly in the savannah zones in the northern part of the country, and early intervention activities were focussed on these areas. From 1975 to 1991, aerial application of insecticide (Temephos) was carried out in rivers and streams close to villages eight districts located in the Comoé River basin (Abengourou, Bouaké, Bouna, Boundiali, Daoukro, Korhogo, Mankono and Séguéla). Intervention focussed on CDTI from 1992 to 2013, although ivermectin distribution was interrupted in most districts due to the civil war in 2003, 2004 and 2007. More recently, CDTi was extended beyond villages in 53 districts located along basins of the Sassandra, Comoé, N’zi-Bandama, Marahoué and Black Volta rivers. MDA programmes for LF using ivermectin combined with albendazole and CDTi for onchocerciasis have been integrated since 2015.

### Implementation of CDTi

According to the National Onchocerciasis Control Programme, ivermectin mass drug administration (MDA) started in Côte d’Ivoire in 1992 in basins of rivers listed above (Sassandra, N’zi-Bandama, Comoé, Marahoué and Black Volta) and in 1998 ivermectin distribution was changed to compile to APOC’s CDTi approach. From 1992 to 2002, some 1,500 villages received at least one round of ivermectin. Villages in areas drained by the Sassandra, Comoé, and N’Zi-Bandama rivers were treated twice per year in 1995 and in 1996 [7]. Community health workers (CHWs) became Community Drug Distributors (CDDs) in 1998 as part of the APOC program [5]. Each year, before each CDTi, CDDs performed a pre-treatment census and updated the community register to show those who had moved away, those who had joined the community, and the newly born. CDDs were trained to use dosing poles to determine the number of tablets of ivermectin to be administered. District health staff and CDDs from targeted villages were trained to conduct CDTi and report any adverse events following treatment to senior district health staff. CDTi was conducted once per year from 1992 in targeted villages listed in 53 districts (S1 Table). The program aimed to cover at least 65% of residents in endemic villages (the coverage goal), but some areas achieved 80% coverage. CDTi implementation was entirely suspended in 13 out of 53 districts located in regions occupied by rebel forces in 2003–4 and in 2007. CDTi was also incomplete in the other 40 districts during this period. MDA was only provided in villages along rivers with fewer than 2,000 inhabitants.

### Ethical clearance

Protocols for epidemiological surveys were reviewed and approved by OCP and APOC scientific committees and included approval by the institutional review board of the Ministry of Health in Côte d’Ivoire. During the first surveys, APOC staff worked together with local staff to make sure that epidemiological surveys were implemented according to validated standard operating procedures [9]. However, all epidemiological surveys were carried out by staff from onchocerciasis control programme of the Ministry of Health.

All participants aged 5 years or above in each site were eligible for inclusion without discrimination on gender, social status, religion or ethnicity. Before being skin snipped, a staff member explained to each participant the purpose and procedures including potential risks and benefits of the study. Participants were allowed to ask any question they may have in relation to the survey. From 2009, all protocols of epidemiological surveys were reviewed and approved by the National Ethics Committee for Research in Côte d’Ivoire.

In brief, oral/ verbal informed consent was obtained from individuals aged 18 years and above. For minors (aged <18 years), written informed consent was obtained from parents or legal guardians, while minors provided oral assent. Due to high illiteracy rate, in some households, oral rather than written informed consent was obtained. The National ethics committee explicitly approved these consent procedures. With the help of the community health workers, participants were informed about the purpose and procedures of the study, including potential risks and benefits in local languages. After explaining the study to the communities and addressing all their questions and comments, the chiefs of all selected villages were asked to put his finger print at the bottom of the participant information sheet. The data were analysed and reported without any directly identifiable information, to maintain anonymity of participants.

### Epidemiological evaluations

#### Selection of sentinel sites

Depending of their accessibility and the size of the district, 2 to 4 villages were surveyed per district during all epidemiological evaluations. Village selection was based on their proximity to the main river (≤ 5 kms) and population (≤ 2,000). Information from local health staff and prior data on onchocerciasis endemicity were also used in the selection process [10]. In most cases the same sentinel villages were repeatedly surveyed. Thus, districts were not systematically or randomly sampled.

#### Parasitological examination

Parasitological surveys were carried out together with clinical examinations between 1975 and 2016 in 42 districts starting with a baseline survey and followed by three follow-up surveys. An additional 11 districts were surveyed for the first time after 2014. Skin snip surveys were conducted in sentinel villages prior to MDA. Briefly, the parasitological examination involved taking two skin snips from each posterior iliac crest using a 2 mm corneoscleral punch (Holth-type). Punches were only used for one person and then sterilized by immersion in ethanol, rinsed in distilled water, and then autoclaved under pressure for 15 minutes.

After collection, the skin snips were placed separately into microtitration plate wells with sterile normal saline solution, and these were viewed by microscopy (40X). The microfilarial load was defined as the arithmetic mean of the MF numbers in two snips from the iliac crests [11]. The first reading was performed after 30 minutes of incubation by experienced parasitologists and a second reading was performed after 24 hr incubation by a senior member of staff to confirm or correct the first reading. Snips were not weighed. For quality control, all positive slides and 10% of the negative slides were re-examined by experienced and qualified independent scientists working with the National Onchocerciasis Control Programme in Côte d’Ivoire.

#### CDTi procedures

Ivermectin (150 μg/kg body weight) was provided in all endemic communities to all residents ≥ 5 years of age apart from pregnant women and people with acute illnesses. All communities located within 5 km from a river and with a population of ≤ 2000 were eligible for CDTi. Larger communities were not treated. From 2003 to 2015 when APOC provided technical support, all epidemiological surveys were carried out 10–12 months after the last CDTi round. Additionally, at the end of each epidemiological survey in sentinel villages, all participants reported as positives and not treated during implementation of annual CDTi were treated.

### Sources of data

Authors reviewed records of the National Onchocerciasis Control Programme and compilation of interviews conducted with current and past programme directors and staff of onchocerciasis control programme.

### Statistical analysis

Results were entered Microsoft Excel spreadsheet and statistical analyses were performed using STATA version 14 (Stata Corporation; College Station, TX, USA). Prevalence and intensity of infection or community MF load (CMFL) were calculated for all districts and compared with baseline data. MF prevalence was calculated as the percentage of participants with MF in skin snips. 95% confidence intervals (CIs) for prevalence were calculated using the Clopper-Pearson exact method. The Chi-squared test was used to compare the differences in prevalence. The National Onchocerciasis Control Programme provided database with CMFL calculation for each site per district, thus for each district median and range was calculated and pairwise comparisons of results by district were performed by the Mann-Whitney test. The authors used geographical information system software ArcGIS version 10.2 (ESRI Inc., Redlands, USA) to draw original maps of onchocerciasis MF prevalence at the district level (S1 Table). Onchocerciasis endemicity was stratified according to World Health Organization guidelines into three levels depending on MF rates in communities: hyperendemic (MF > 60%), hypoendemic (MF <35%) and mesoendemic onchocerciasis (MF between 35% and 60%) [12].

## Results

### History of interventions conducted for onchocerciasis control

#### Onchocerciasis control using larvicides

During the OCP period (1974–1991), the rivers in all endemic districts were sprayed once a week with larvicidal insecticide. During the APOC period (2003–2015 with an interruption between 2004 and 2007), only hyper and meso-endemic districts were treated by CDTi according to WHO recommendations [13]; CDTi was not provided in hypo-endemic areas.

Use of larvicides started in 1975 in villages in 8 districts (Abengourou, Bouaké, Boundiali, Daoukro, Katiola, Korhogo, Mankono and Séguéla) located along the Feredougouba river, a tributary of Sassandra river [14]. This intervention included weekly aerial application of insecticide (temephos) to the riverine breeding sites of the vector *Simulium damnosum s*.*l*. The spraying program ended in 1991.

#### Reduction in MF prevalence and density after aerial application of insecticide

**Fig 1:**
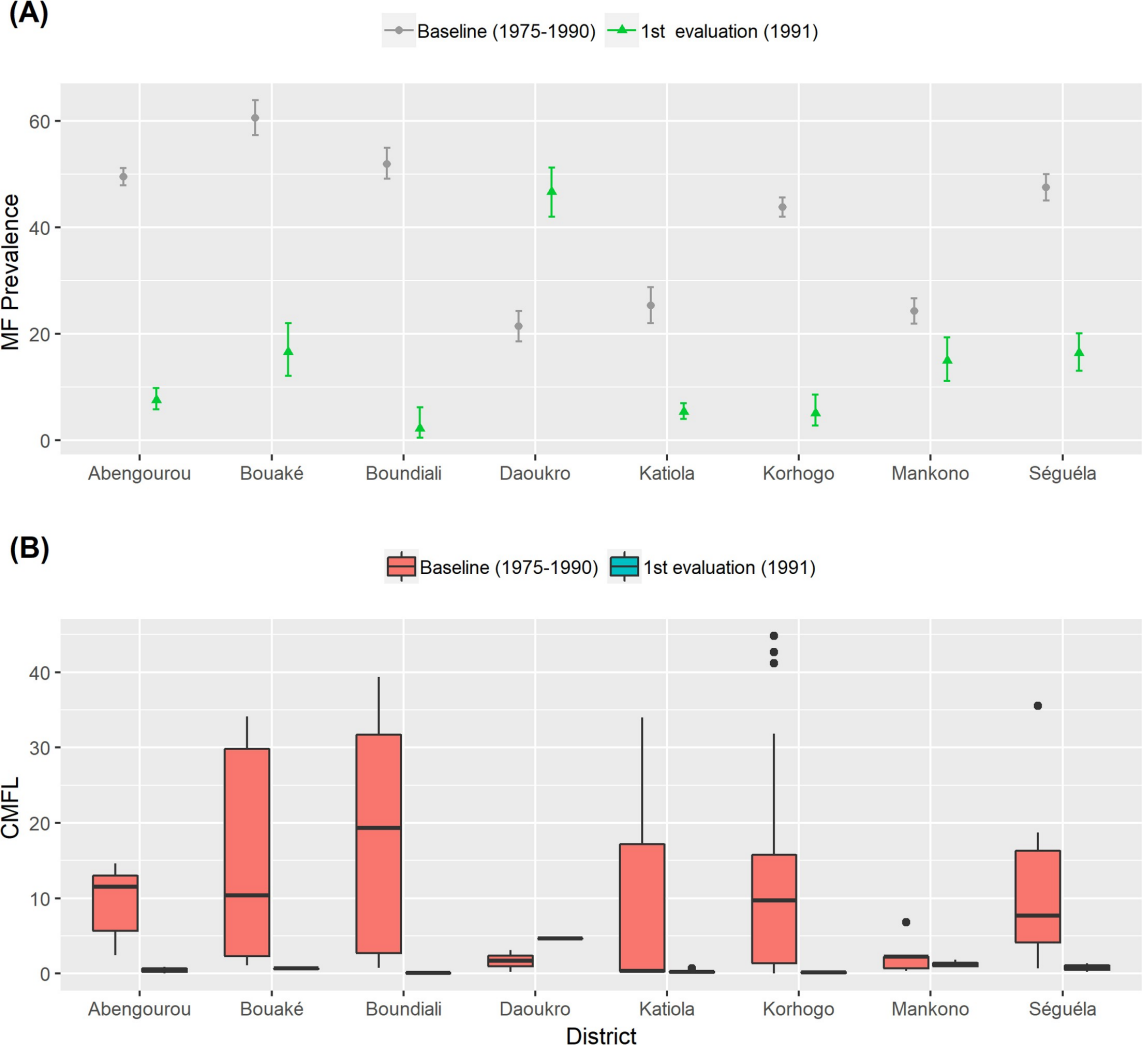
Results of 1975–1991 onchocerciasis skin snip microfilaria prevalence and density by district in Côte d’Ivoire. (A) MF prevalence, (B) MF density.

Fig 1A and 1B presents onchocerciasis MF prevalence and density data by district at baseline and reductions observed after weekly aerial insecticide spraying in 8 endemic districts, in Côte d’Ivoire (1975–1991). This intervention resulted in significant reductions in MF prevalence relative to baseline proportions in 7 out of 8 surveyed districts (P < 0.001). Overall MF prevalence dropped significantly [from 43.5 (95%CI 42.6–44.3)% to 13.9 (95%CI 12.8–15.1)%, P< 0.001]. The overall reduction of MF prevalence was 68.1%. CMFL decreased significantly in 7 out of 8 surveyed communities by 95.2% from 9.24 (range 0.05–44.8) MF/snip to 0.44 (range 0–4.64) MF/snip (U = 5.290; P<0.001). Surprisingly, in Daoukro district, there was a significant increase in MF prevalence [from 21.4 (95%CI 18.6–24.3) to 46.7% (95%CI 42.0–51.2)%, P<0.001] and an increase in CMFL [from 1.69 (range 0.23–3.14) MF/snip to 4.64 MF/ snip; P = 0.221]. In the endemic districts surveyed, reductions in MF prevalence ranged from 38 to 95%, and CMFL reductions ranged from 40 to 99%.

### CDTi coverage from 1992 to 2015

Larger villages were not treated with ivermectin between 1992 and 2014. Annual MDA with ivermectin plus albendazole was initiated in districts co-endemic for onchocerciasis and LF in 2015 while districts endemic for onchocerciasis only were treated from 1992 to 2014.

**Fig 2:**
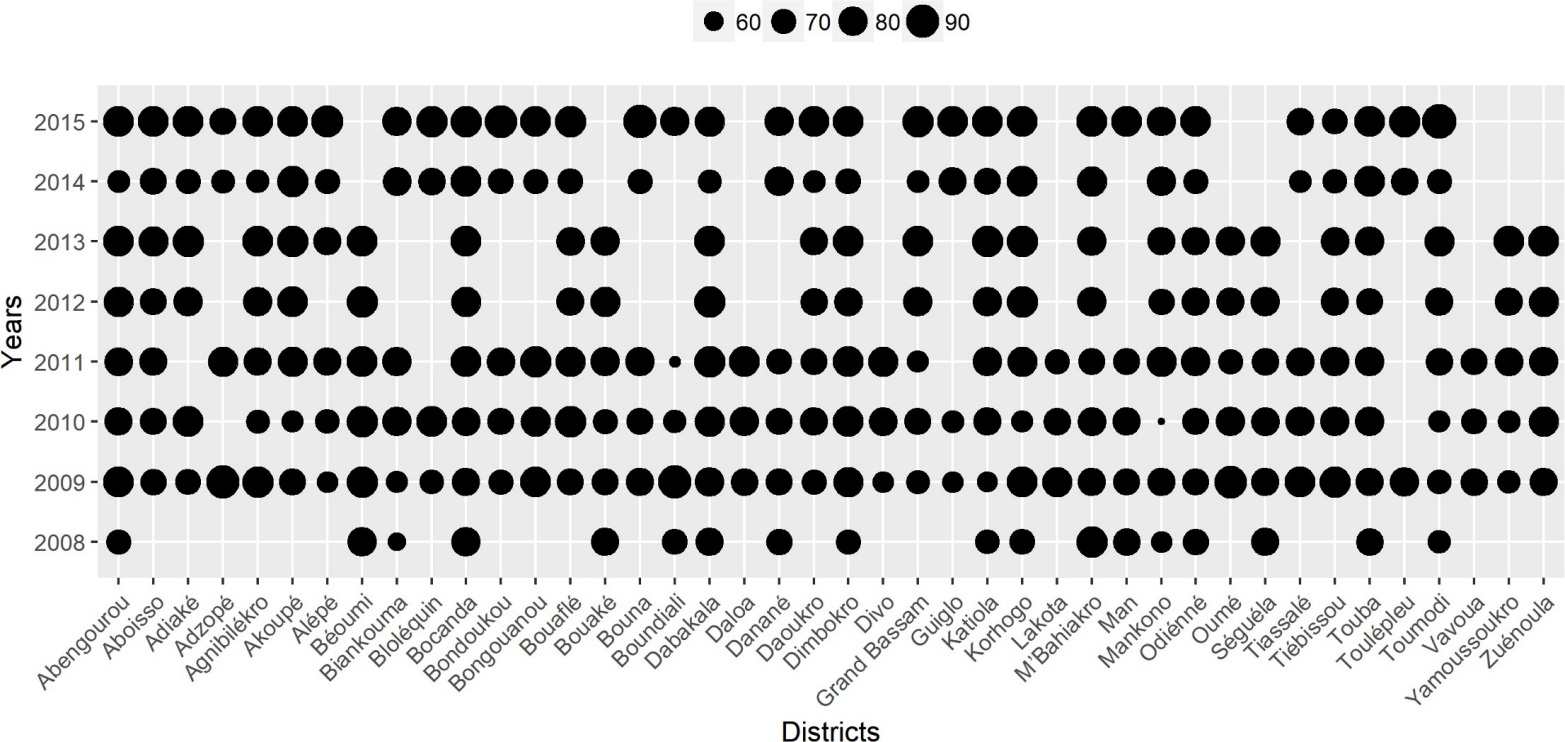
Proportion (%) of eligible people treated against onchocerciasis (therapeutic coverage) in endemic districts from 2008 to 2015.

Ivermectin distribution was performed in limited areas in 1992. From 1995 to 1998, 350,000 to 370,000 persons were treated per year. These populations were located along the basin of Sassandra, Black Volta, Comoé, N’Zi-Bandama and Bou-Bandama rivers. From 2003 to 2004, and in 2007 due to the instability and the civil war, MDA was not implemented in 13 out of 28 districts belonging to the Western, Northern and Central regions (Man, Korhogo, Bouaké, Odiénné, Bouna) occupied by rebels. CDTi re-started in 2005. From 2008 to 2014 CDTi was carried out continuously in only 11 districts (Abidjan, Aboisso, Dabou, Gagnoa, Grand Lahou, Sakassou, Sikensi, Sinfra, Tabou, Touleupleu, Zouan-Hounien) and incomplete in 15 districts. S2 Table presents the number of people eligible and treated for onchocerciasis from 2008 to 2015 in endemic districts. Therapeutic coverage was at least 65% between 2008 and 2013 with the following exceptions: Biankouma and Mankono districts in 2008, Divo, Guiglo and Zouan-Hounien districts in 2009, Mankono, Korhogo, Bangolo and Akoupé districts in 2010, and Boundiali districts in 2011 (Fig 2). The 65% coverage target was not achieved in Gueyo, Sassandra, Soubré and Tabou districts in 2014. From 2012 to 2016, the therapeutic coverage for elimination (80%) [10] was achieved in most endemic districts. It is important to emphasize that ivermectin distribution implemented before 2015 did not cover larger villages with more than 2,000 inhabitants as recommended in CDTi strategy. However, since 2015 CDTi has been integrated and extended to entire districts co-endemic for LF and onchocerciasis meaning that ivermectin and albendazole are now given to all eligible people in co-endemic districts for LF and onchocerciasis (and not only in villages with less than 2,000 inhabitants).

In 2015 to 2016, 100% geographical coverage was reached for all districts co-endemic for LF and onchocerciasis, and in districts endemic only for onchocerciasis, and 80% therapeutic coverage was achieved in most districts during this period.

### Baseline onchocerciasis MF prevalence prior to all interventions

**Fig 3:**
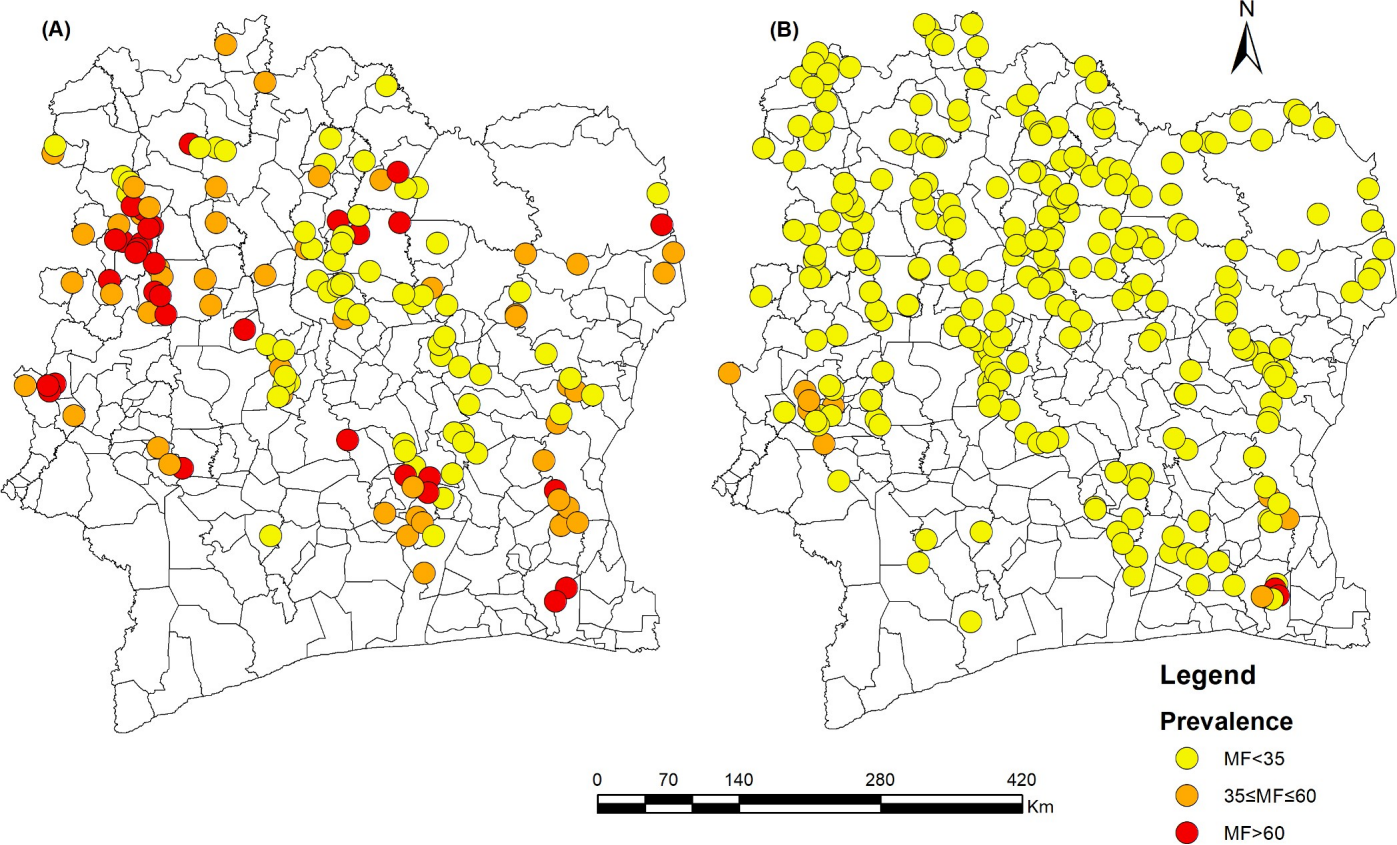
Baseline onchocerciasis MF prevalence of onchocerciasis assessed by microfilaria detection in skin snips. (A) 1975–1991 Baseline evaluation prior to aerial spraying; (B) 1992–2016 baseline evaluations of MF prevalence in additional districts during CDTi.

Fig 3 summarise the results of the baseline of onchocerciasis MF prevalence, in 47 endemic districts from 1975 to 2016.

Baseline MF prevalences between 1975 and 1991 from 8 districts (based on skin snip data from 26,507 people varied from 3.9% (95% CI 2.0–6.7) to 85.3% (95%CI 79.4–89.6). From 2008 to 2014, baseline survey was carried out in 11 additional districts located in the forest region. The overall MF prevalence for these 11 districts was 7.0% (95%CI 6.4–7.8) and the CMFL was 0.08 (range 0–4.01) MF/ snip (S3 Table). No epidemiological evaluation was carried out in these 11 districts. In total, 47 districts were surveyed from 1975 to 2016 (6 out of 53 districts missing baseline were excluded from analysis). Based on skin MF prevalence data presented in Fig 3A, three of 36 districts in the (forest region) were classified as meso-endemic (Alépé, Danané, Soubré), and 33 districts were hypo-endemic. In the savannah area, one of seven districts was hyperendemic, five were meso-endemic, and one was hypo-endemic. In the forest area, baseline surveys were carried out in 16 districts. Six were found to be hyperendemic, 6 were meso-endemic, and 4 were hypo-endemic. In the pre-forest area (transition from forest to savannah) in central and Eastern Côte d’Ivoire, 3 of 13 districts were meso-endemic and 10 were hypo-endemic. The overall MF prevalence (means of MF prevalence rates recorded in districts belonging to different ecological zones) recorded in savannah, forest and pre-forest regions was 47.4% (95% CI 46.6–48.1), 46.8 (95% CI 46.1–47.5)% and 31.3 (95% CI 30.6–31.9)%, respectively. Onchocerciasis MF prevalence was significantly lower in pre-forest regions compared to savannah (p< 0.001) and forest regions (p< 0.001).

### Baseline onchocercal MF density before all interventions

S2 Table presents baseline median MF densities for 36 districts. The median value for these districts was 6.69 (range 0–123.3) MF/snip. The overall density of MF was 0.08 (range 0–4.01) MF/snip for 11 districts in forest region that had baseline surveys between 2008 and 2014. CMFL levels in districts located in forest, savannah and pre-forest regions were 11.52 (range 0.02–118.05) MF/snip, 8.44 (range 0–123.3) MF/snip and 2.27 (0.01–81.48) MF/snip, respectively. MF density was significantly lower in pre-forest regions compared to savannah (p = 0.019) and forest regions (p = 0.003).

### Reductions in MF prevalence and density after ivermectin distribution

**Fig 4:**
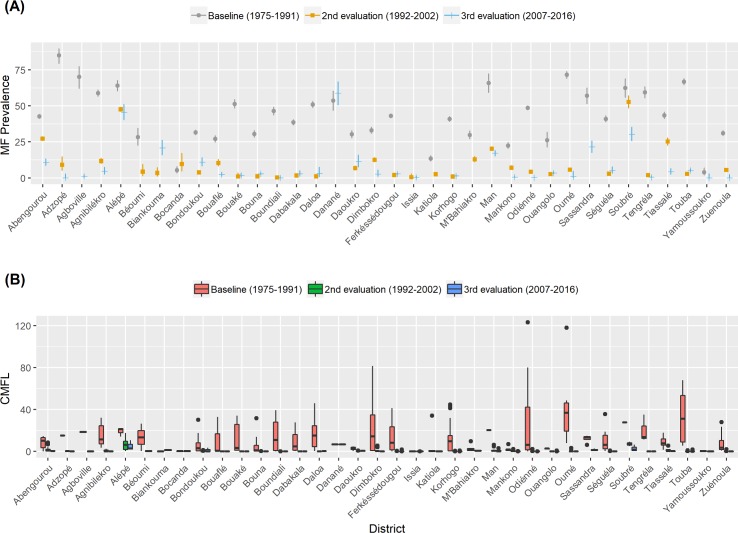
Results of 1975–2016 onchocerciasis skin snip microfilaria prevalence and density by district in Côte d’Ivoire. (A) MF prevalence, (B) MF density.

Fig 4A and 4B presents data on onchocerciasis MF prevalence and density by district at baseline and after the second and third epidemiological evaluations were conducted after periods of annual CDTi. The first survey (1992–2002) and the second survey (2007–2016) covered 36 endemic districts. Results from the first repeat survey showed that MF prevalence decreased in 35 out of 36 surveyed districts following ivermectin distribution. The overall MF prevalence dropped significantly by 75.7% [from 41.6% (95%CI 41.2–42.0) to 10.1% (95%CI 9.9–10.3), P< 0.001]. Percent reductions in MF prevalence by district varied from 15.6 to 99.4% (S2 Table). Bocanda district was an exception where the MF prevalence increased from 5.3% (95%CI 3.5–7.7) to 9.6% (95% CI 4.7–17.0).

The second follow up epidemiological evaluation also documented reductions in MF prevalence in most districts. The overall MF prevalence dropped by 86.5% from baseline [from 41.6% (95%CI 41.2–42.0) to 5.6% (95%CI 5.4–5.9), P< 0.001)]; percent reductions varied from 80 to 100% in 23 districts and between 70–80% in 4 districts. There was a significant decrease in overall MF prevalence between the first and second follow up surveys [from 10.1% (95%CI 9.9–10.3) to 5.6% (95%CI 5.4–5.9), P< 0.001].

Based on comparisons of baseline and first follow-up surveys, the reduction in MF prevalence after weekly aerial insecticide spraying (16 years of interventions) was significantly lower than after ivermectin distribution (15 rounds of CDTi) (68.1% vs 75.7% reduction) (P< 0.001).

The first and second follow up surveys also documented dramatic reductions in CMFL compared to baseline [from 6.69 (range 0–123.3) MF/snip to 0.05 (range 0–17.62) MF/snip after weekly aerial larvicide spraying and from 6.69 (range 0–123.3) MF/snip to 0.01 (0–10.71) MF/ snip after CDTi implementation, respectively]. CMFL values decreased in all districts except in Bocanda where a slight increase was recorded (from 0.17 MF/snip to 0.3 MF/snip). CMFL values were 99.3% and 99.8% lower than baseline at the time of the first and second follow up surveys, respectively. These reductions were superior to those achieved by aerial spraying of insecticides in the 1970s. Reductions in CMFL from baseline by district in the first and second follow up surveys ranged from 70–100% and 81–100%, respectively.

### Impact of civil war and interruption of CDTi on prevalence and density of onchocerciasis MF (2005–2006; 2008–2015)

Although high coverage rates were reported from most endemic districts in most years, results presented in Fig 2 and S3 Table show that many districts missed one or more rounds of MDA between 2005 and 2015. MDA was continuously implemented in only 11 districts after 2007. For example, Lakota district received MDA from 2009 to 2011 but missed 5 rounds (2008, 2012, 2013, 2014 and 2015 rounds). Some districts were assessed during the first follow up survey program but not in the second, and vice versa. This makes comparisons over time difficult in some cases.

Due to the civil war and instability, CDTi was not implemented in 2003, 2004, and 2007. Also, as mentioned above, CDTi was only intermittently provided in most districts after 2008. Data from districts surveyed during the first and second follow up surveys (Fig 4A) showed an increase in MF prevalence in 12 districts (Biankouma, Bondoukou, Bouaké, Bouna, Dabakala, Daloa, Daoukro, Ferkéssédougou, Korhogo, Ouangolo, Séguéla, Touba). On the other hand, MF prevalence and CMFL decreased between the two follow up surveys in 13 endemic districts (Abengourou, Adzopé, Alépé, Dimbokro, Ferkéssédougou, Issia, Man, Mankono, Odiénné, Soubré, Tengréla, Tiassalé, Zuenoula) despite gaps in MDA in nine of them. Only four of these 13 districts were continuously treated since 2008 (Abengourou, Dimbokro, Mankono, Odiénné**).** In Bouaké and Korhogo districts, the MF rate was <1% in the first follow up survey carried out between 1992 and 2001 but > 1% after 2002. According to World Health Organisation-APOC guidelines [9], this increase meets the criterion for a recrudescence.

## Discussion

OCP was set up in 1974 with the objective to control the disease as a public health problem rather than to achieve elimination of the infection and transmission. During OCP period, control of onchocerciasis was planned to be achieved by weekly aerial application of insecticide (temephos) to the riverine breeding sites of the vector *Simulium damnosum s*.*l*. The original OCP area included most of West African countries such as Burkina Faso, Mali, Côte d’Ivoire, Ghana, Togo, Benin and Niger. In Côte d’Ivoire, OCP success in Côte d’Ivoire was illustrated by the significant drop recorded after more than 10 years of aerial insecticide spraying. Onchocerciasis MF prevalence dropped significantly from 43.1% (95%CI 42.6–44.3) to 13.9% (95%CI 12.8–15.1) as well as the CMFL [from 9.24 (range 0.05–44.8) MF/snip to 0.44 (range 0–4.64) MF/snip].

After OCP interventions, prevalence of onchocerciasis dropped steadily but was less than 5% in only one out of 8 districts surveyed after the cessation of vector control by 1991. Onchocerciasis MF prevalence was above 10% in 6 out of 8 districts. Vector control contributed to reduce significantly the prevalence of onchocerciasis in most OCP countries. However, prevalence of infection in the human population never dropped to zero despite 10 years of intervention, and they were not below the level at which it may represent the transmission breakpoint in 7 out of 8 districts [10]. A recent epidemiological modelling study concluded that the risk of recrudescence depended on the pre-control endemicity level as an indicator of the local potential of transmission [15]. Though in some of our districts the pre-control endemicity levels were high, the country has now a chance for elimination of this disease knowing that since June 2017 MDA is implementing annually in the entire country [16]. This was not observed probably because most of the study villages may have been subject to vector reinvasion and the programme experienced short periods of suspension of aerial larvicides application. Furthermore, suspension of larviciding during some periods may have contributed to increase productivity of blackfly breeding sites which may have contributed to maintain vector densities at a level that could sustain transmission of *O*. *volvulus* as previously demonstrated [17].

After years of aerial insecticide spraying, control of onchocerciasis was complemented by ivermectin mass treatment of the at-risk population after Merck & Co. Inc. agreed to donate ivermectin in 1987 [18]. This new strategy led by APOC was based on the implementation of CDTi which was successfully conducted until 2007. With APOC creation in 1995, a strong partnership was set up with onchocerciasis endemic countries which has helped these countries to successfully extend CDTi coverage from 1.5 million in 1997 to over 112 million people in 2014, reaching over 180 000 communities in Africa [19]. This enormous achievement in control of onchocerciasis in Africa was materialized by growing evidence that ivermectin alone could be used to eliminate infection and reduce transmission to levels at which treatment with ivermectin could be safely stopped after more than 10 rounds of CDTi implementation. In Côte d’Ivoire, after at least five rounds of annual ivermectin distribution, the second and the third epidemiological evaluations in 36 endemic districts, revealed significant decreases in onchocerciasis MF prevalence and CMFL.

Indeed, before ivermectin distribution started in 1992, the overall community prevalence of *O*. *volvulus* MF in the skin was 41.6% (range 3.9% to 85.0%). After 8–10 years of treatment, the prevalence had fallen to less than 1% in 2 districts and remained above 5% in 15 endemic districts. In view of previous experiences with onchocerciasis elimination in West Africa, these reductions are significant but lower than those observed in Senegal and Mali where the prevalence of MF had fallen below 1% in most of the villages after implementation of 10 rounds of CDTi [20]. However, the reduction observed in some endemic districts of Côte d’Ivoire may be considered to be important, because it occurred after implementation of a minimum of 5 rounds of CDTi. Indeed, repeated and continuous annual distribution of ivermectin helped to prevent the onset of new cases and reduce transmission [21] with the goal of reducing onchocerciasis prevalence to a level that cannot sustain transmission. Similar studies aimed to determine onchocerciasis MF prevalence and CMFL have been reported in Sierra Leone and Cameroon after repeated annual treatment with ivermectin [22,23]. Our findings are similar to those recently reported in Sierra Leone where skin MF prevalence were still between 10 and 30% after implementation of 5 continuous MDA rounds with ivermectin [24].

This paper has emphasized that efforts to control onchocerciasis in Côte d’Ivoire have made a significant impact despite various challenges. Although civil conflict has hampered onchocerciasis control programs in Côte d’Ivoire and neighbouring countries, we are optimistic about the future; improved political stability should facilitate regular distribution of ivermectin that will slowly reduce infection prevalence and intensities and shrink the map. Indeed, studies conducted recently in several meso and lower-hyper-endemic settings in Mali, Senegal [25] and Nigeria [26] have shown that repeated distribution of ivermectin alone can interrupt *O*. *volvulus* transmission, and these results provide a proof of principal for Côte d’Ivoire’s program.

Data from Côte d’Ivoire showed that disruption in CDTi related to the civil war had an effect on onchocerciasis rates or CMFL. The overall MF prevalence and the CMFL increased from the second to the third evaluation in 10 endemic districts. This may be firstly due to the absence of MDA implementation in 2003, 2004 and 2007 in endemic districts from Western, Northern and Central regions occupied by rebels, and secondly, from 1992 to 2014, only 10 out of 36 districts surveyed for onchocerciasis were continuously treated with ivermectin meaning that MDA was not carried out continuously in the other 26 districts. Finally, before 2015, in Côte d’Ivoire ivermectin distribution never reached villages with more than 2,000 inhabitants even if they are close to basins of rivers hosting breeding sites of black flies. These weaknesses may have contributed to the increase of MF prevalence and CMFL in these 10 districts.

One of the great achievements of Côte d’Ivoire Onchocerciasis control programme is that MDA coverage was often above the target required for control and for elimination in regions controlled by the elected government where MDA implementation was not suspended. The challenge now will be to deliver ivermectin with high compliance to all endemic areas in the country because it was done previously at small scale. As MDA will now be carried out at district level in all districts co-endemic for LF and onchocerciasis, the country may be faced to challenges such as hard to reach areas, migratory populations, local health systems deficiencies (e.g. at the level of CDD). Our findings showed that reaching the effective coverage threshold for elimination (>65%) is key for accelerating elimination of preventive chemotherapy diseases such as LF and onchocerciasis. Our results corroborated results of recent study carried out in Cameroon where despite more than 15 years of mass treatment, microfilaridermia prevalence did not decrease [27]. In this study the persistence of the transmission of *O*. *volvulus* was favoured by the low adherence of targeted communities to treatment because only 40 to 54% of people from the study areas confirmed having taken five treatments during the last five years [27]. Thus, as demonstrated recently in Mali and Nigeria [25,26], if CDTi continues to be well implemented in Côte d’Ivoire, this may lead to interruption of onchocerciasis transmission the coming years. A recent APOC review concluded that that effective ivermectin distribution can lead to elimination of onchocerciasis if CDTi is properly implemented as demonstrated in many countries [28]. Côte d’Ivoire would like to contribute its own success stories to this narrative, but this will take time, effort, and sustained support from within the country and from international partners.

Despite important findings presented previously, our study has several limitations. The data presented are government surveillance data and were not collected as part of a systematic study. For example, epidemiological assessments were not carried out in the same years in all districts. Not all endemic areas received ivermectin, and surveys did not sample localities with populations greater than 2,000. Despite these limitations, the surveillance data are valuable, because they demonstrate the impact of interventions during different periods over the past 40 years in Côte d’Ivoire. The elimination effort will require more and better data. Current WHO elimination guidelines [29] recommend starting with a situation analysis with updated surveys to confirm the current status of each district. Districts with MF rates <1% should have entomological evaluation in order to detect residual transmission during at least one transmission season before confirming interruption of transmission [29].

In conclusion, despite the limitations and weaknesses of the available data, this large body of data collected over many years documents very significant impact of interventions (vector control and CDTi) by the national onchocerciasis control programme despite challenges. OCP and APOC support were essential contributors to this success. However, our study also showed clearly that Côte d’Ivoire has still a major onchocerciasis problem due mainly to the civil war and instability of the country, lack of support from international donors resulting in the irregularity of MDA implementation for onchocerciasis. Now that the country has a unique programme for all the five preventive chemotherapy diseases and has started since 2015 MDA national coverage for LF and onchocerciasis, we are confident that great progress will be made to move from control to elimination of the disease. However, where onchocerciasis transmission will persist despite successful MDA implementation, the country will need to carry out alternative strategies such as twice annual distribution of ivermectin, doxycycline test and treat and/or ground larviciding.

## Supporting information

S1 Checklist STROBE checklist. (DOC)

S1 Table GPS coordinates for surveyed villages. (DOCX)

S2 Table Results of annual MDA rounds carried out continuously for onchocerciasis elimination in Côte d’Ivoire from 2008 to 2015. (XLSX)

S3 Table Results of onchocerciasis microfilaria prevalence and density by district in Côte d’Ivoire. (DOC)
