# Supplementary material for: Update on the current status of onchocerciasis in Côte d’Ivoire following 40 years of intervention: Progress and challenges
Source: PLoS Negl Trop Dis. 2018 Oct 23;12(10):e0006897. doi: 10.1371/journal.pntd.0006897 (PMC6214569; doi:10.1371/journal.pntd.0006897)
Supplement: S1 Table — (DOCX) [file pntd.0006897.s002.docx]

**S1 Table**: GPS coordinates for surveyed villages

- **Baseline surveyed (1975-1991)**

| **Id** | **Villages** | **Latitude** | **Longitude** |
| --- | --- | --- | --- |
| 1 | ABRADINOU | 6.20611 | -3.59972 |
| 2 | ADAOU | 6.28333 | -4.88333 |
| 3 | AGUIBRI | 6.93306 | -5 |
| 4 | AHININKRO | 6.51667 | -3.65 |
| 5 | AHUA | 5.78333 | -4.81667 |
| 6 | AKA-KOMOEKRO | 7.45 | -3.53306 |
| 7 | ALEY | 6.96667 | -5.5 |
| 8 | ALOSSO | 5.65 | -3.55 |
| 9 | AMOUKOUKRO | 7.11667 | -3.63306 |
| 10 | ANDIANOU | 7.01667 | -4.46667 |
| 11 | AOWATI | 6.11667 | -4.96667 |
| 12 | ASSADIENKRO | 6.85 | -4.35 |
| 13 | ASSEMANU | 6.78306 | -3.75 |
| 14 | ASSEREKRO | 6.61667 | -4.85 |
| 15 | AWAHIKRO | 8.28306 | -3.96667 |
| 16 | BAADALA | 8.09972 | -7.26667 |
| 17 | BADA | 8.13306 | -5.51667 |
| 18 | BAGOZRA | 7.36667 | -6.08306 |
| 19 | BAGRO | 6.71667 | -6.96667 |
| 20 | BANANKORO | 8.43333 | -6.23333 |
| 21 | BAYOLA | 8.36667 | -7.95 |
| 22 | BEDIAKRO | 6.45 | -4.65 |
| 23 | BENIASSO | 10.48306 | -6.58306 |
| 24 | BEREMA | 8.4 | -6.76667 |
| 25 | BISSIDOUGOU | 8.66667 | -5.86667 |
| 26 | BLEKOUM | 6.36667 | -3.53306 |
| 27 | BLEMPLO | 8.08306 | -5.4 |
| 28 | BOKOBA | 8.86667 | -7.23306 |
| 29 | BONI-ANDOKRO | 6.73306 | -4.9 |
| 30 | BOROTOU | 8.73333 | -7.5 |
| 31 | BOUNTOU | 8.49306 | -7.18056 |
| 32 | BOUROUNON | 7.73306 | -6.11667 |
| 33 | CAMP. SOULEMANEDOUGO | 8.25 | -4.83306 |
| 34 | DABALA | 8.41667 | -7.15 |
| 35 | DANANGORO | 7.18306 | -7.93306 |
| 36 | DANTOGO | 7.81667 | -6.21667 |
| 37 | DIEOULIZRA | 7.48306 | -6.01667 |
| 38 | DIKODOUGOU | 8.38306 | -5.76667 |
| 39 | DOUMBA | 8.97139 | -7.33194 |
| 40 | FANATOKODA | 8.33306 | -5.58306 |
| 41 | FARAKO | 9.31278 | -7.5 |
| 42 | FARANDOUGOU | 9.21667 | -6.66667 |
| 43 | FETEKRO | 7.81667 | -4.68306 |
| 44 | FODIOLOKAHA | 9.45 | -5.35 |
| 45 | GALEGOUA | 6.23306 | -4.83306 |
| 46 | GANSE | 8.62222 | -3.91472 |
| 47 | GBAGBA-SIRAKORO | 8.08306 | -4 |
| 48 | GBANDO | 9.56667 | -6.66667 |
| 49 | GBANGBO-TIEMELEKRO | 7.03333 | -4.55 |
| 50 | GBANHALA | 9.51867 | -8.12222 |
| 51 | GBANTOPLEU | 7.45 | -8.36667 |
| 52 | GBELEBAN | 9.585 | -8.10333 |
| 53 | GBOLY CARREFOUR(CAMP | 8.16667 | -4.61667 |
| 54 | GHANASSO | 8.36667 | -5.53306 |
| 55 | GNENINFOLOKAHA | 8.56667 | -5.61667 |
| 56 | GOUENDIEDOUGOU | 9.16111 | -7.45556 |
| 57 | GUESSO-BONDOSSO | 8.28306 | -5.66667 |
| 58 | GUIENDANA | 9.21167 | -4.87917 |
| 59 | GUINGUERENI | 9.54111 | -6.58586 |
| 60 | KAKOLOGO | 10.15 | -6.23306 |
| 61 | KAMALA | 8.45 | -2.68306 |
| 62 | KANGAKRO | 7.4 | -3.46667 |
| 63 | KAVAKA | 7.60194 | -6.09528 |
| 64 | KEBEKO | 9.6 | -6.9 |
| 65 | KOBADARA (POINT VII) | 9.20361 | -4.98194 |
| 66 | KOBALA | 9.01667 | -7.31667 |
| 67 | KOGUINA | 6.66667 | -4.56667 |
| 68 | KOLOKAHA | 8.9 | -5.03306 |
| 69 | KOLON | 8.28333 | -7.21667 |
| 70 | KOMOE-N'GOUA | 7.2 | -3.6 |
| 71 | KOUASSIBILEKRO | 7.73306 | -3.73306 |
| 72 | KOUEZRA | 6.11667 | -6.18306 |
| 73 | KOUROUKORO | 7.95 | -6.41667 |
| 74 | KOUROUKOUNA | 9.28305 | -5.2 |
| 75 | KROKOKRO | 6.63306 | -4.76667 |
| 76 | LAMEKAHA | 9.35 | -5.04972 |
| 77 | LAMOUEDOUGOU | 8.79917 | -7.85 |
| 78 | LAOUREBO | 6.5 | -4.78306 |
| 79 | LERABA-GARE | 10.11667 | -5.15306 |
| 80 | LISSOLO | 8.06667 | -4 |
| 81 | LOMO NORD | 6.65 | -4.98306 |
| 82 | LONGO | 8.96667 | -5.4 |
| 83 | LONIENE | 8.31667 | -4.75 |
| 84 | MAMORODOUGOU | 8.71667 | -7.33306 |
| 85 | MAMOUYA | 8.88306 | -7.53306 |
| 86 | MANGWA-OKOUKROU | 8.05 | -5.53333 |
| 87 | MASSADOUGOU | 9.05 | -7.41667 |
| 88 | MOOFOUE | 6.11667 | -4.73306 |
| 89 | NABEDJAKAHA | 8.78306 | -5.53306 |
| 90 | NAFANA-SIENSO | 9.26389 | -7.43889 |
| 91 | NAMBAYEREDOUGOU | 8.71667 | -4.7 |
| 92 | NAMOURGUEKAHA | 9.41667 | -5.7 |
| 93 | N'GAPIE | 9.56667 | -6.81667 |
| 94 | N'GORONDOUGOU | 8.38806 | -7.61917 |
| 95 | NIANDA | 5.53306 | -3.65 |
| 96 | NIDROU | 6.9 | -7.18333 |
| 97 | NIENESSO | 9.21667 | -7.4 |
| 98 | NIMPLEU | 7.46667 | -8.1 |
| 99 | OUANGUI | 7.36667 | -3.31667 |
| 100 | OUASSIKO | 8.75 | -7.56667 |
| 101 | OUATTARADOUGOU | 8.66667 | -5.81667 |
| 102 | OUEREGUEKAHA | 8.46667 | -5.3 |
| 103 | PETONKAHA | 8.8 | -5.4 |
| 104 | PIEYE | 8.88306 | -2.7 |
| 105 | POHIZRA I | 7.45 | -6.06667 |
| 106 | POTOSSOU | 7.28306 | -4.41667 |
| 107 | PRANOA | 6.86667 | -4.98306 |
| 108 | SAMATIGUILA | 8.81667 | -5.88306 |
| 109 | SANANKOROBA | 8.85 | -7.26667 |
| 110 | SAWA | 6.31667 | -5.16667 |
| 111 | SETOUMOU | 8.9 | -6.66667 |
| 112 | SIOBA | 7.4 | -8.15 |
| 113 | SOROTONA | 8.08306 | -7.11667 |
| 114 | SOUROUMANA | 8.16667 | -6.71667 |
| 115 | TAFOLO | 8.26667 | -5 |
| 116 | TAGADI (5129) | 8.63306 | -2.6 |
| 117 | TANWARA-(DJERO) | 9.65083 | -5.65111 |
| 118 | TCHEBROU | 8.35 | -5.55 |
| 119 | TENIAKRO | 6.95 | -4.46667 |
| 120 | TIEBEKAHA | 8.91667 | -5.58333 |
| 121 | TIEBISSOU | 7.71667 | -4.66667 |
| 122 | TIEKORODOUGOU | 9.03333 | -7.26472 |
| 123 | TIEKORONIDOUGOU | 8.66667 | -7.36667 |
| 124 | TIEN-OULA | 6.75 | -7.08333 |
| 125 | TOFESSO | 7.76667 | -6.06667 |
| 126 | TONTONOU - KOFFIKRO | 6.55 | -4.91667 |
| 127 | TOUMBO-YAGA | 8.53333 | -3.45 |
| 128 | TRIPOUNGO | 9.3125 | -5.75 |
| 129 | TYEIFLA | 7.53272 | -6.05406 |
| 130 | VIALADOUGOU | 8.54028 | -7.21861 |
| 131 | VONKORO | 9.15972 | -2.73611 |
| 132 | VREHENFLA | 7.35 | -6.11667 |
| 133 | WA | 7.45 | -8.16667 |
| 134 | WEBASO | 8.25 | -7.16667 |
| 135 | WENGTOULO | 8.26667 | -7.6 |
| 136 | WINDOU-SOKORO | 8.63333 | -7.38333 |
| 137 | YAMAUEKRO | 7.88306 | -4.63306 |
| 138 | YAPEKRO | 7.61667 | -4.5 |
| 139 | YEKOLO | 8.18306 | -4.91667 |
| 140 | YEREYERE | 6.23306 | -3.45 |
| 141 | YOMANKRO | 7.51667 | -3.51667 |
| 142 | ZANAKAHA | 8.71667 | -5.55 |
| 143 | ZANOUKORO | 7.55 | -4.31667 |
| 144 | ZONOKOI | 6.43306 | -3.61667 |

- **First and second survey (1992-2016)**

| **Id** | **Villages** | **Latitude** | **Longitude** |
| --- | --- | --- | --- |
| 1 | ABOKOSSO | 7.6 | -3.6 |
| 2 | ABOUDE DADIER | 5.934138889 | -4.49775 |
| 3 | ABRADINE II | 6.20611 | -3.59972 |
| 4 | ABRADINOU | 6.20611 | -3.59972 |
| 5 | ACHIEKOI | 5.640777778 | -3.933944444 |
| 6 | ADJAME | 7.11806 | -7.11917 |
| 7 | AFERI | 7.75 | -3.51944 |
| 8 | AHININKRO | 6.51667 | -3.65 |
| 9 | AKA-KOMOEKRO | 7.45 | -3.53306 |
| 10 | Alley | 7.623138889 | -5.856944444 |
| 11 | ALOSSO | 5.65 | -3.55 |
| 12 | AMANIKRO | 7.68306 | -6.15 |
| 13 | AMAPOKOUASSIKRO | 7.71667 | -3.71667 |
| 14 | ANANGUIE/Belle ville | 6.073666667 | -4.471222222 |
| 15 | ANGOUAKRO | 7.26667 | -3.6 |
| 16 | AOWATI | 6.11667 | -4.96667 |
| 17 | ASSADIENKRO | 6.85 | -4.35 |
| 18 | ASSEMANOU (0383) | 6.78306 | -3.75 |
| 19 | ASSEMANU | 6.78306 | -3.75 |
| 20 | ASSEREKRO | 6.61667 | -4.85 |
| 21 | ATEHOU | 5.648444444 | -4.256166667 |
| 22 | ATIMABO | 7.75 | -3.83306 |
| 23 | AWAHIKRO | 8.28306 | -3.96667 |
| 24 | AWONWONKRO | 5.51944 | -3.58889 |
| 25 | BADA | 8.13306 | -5.51667 |
| 26 | BADA | 8.486777778 | -6.46625 |
| 27 | BADALA-ODIENNE | 8.5 | -5.58333 |
| 28 | BADENOU | 9.90944 | -5.85639 |
| 29 | BADENOU II | 10 | -5.76667 |
| 30 | BAGOZRA | 7.36667 | -6.08306 |
| 31 | Baïssa Carrefour | 6.048861111 | -6.671055556 |
| 32 | BAKOMIVOGO | 10.25667 | -5.25167 |
| 33 | Bakomivogo | 10.61111111 | -5.203111111 |
| 34 | BAMABALOUMA | 7.8515 | -6.004555556 |
| 35 | BAPOLKAHA | 9.2 | -5.56667 |
| 36 | BAWE | 9.58167 | -4.15333 |
| 37 | BENIASSO | 10.48306 | -6.58306 |
| 38 | BISSIDOUGOU | 8.66667 | -5.86667 |
| 39 | BLAMA | 6.994055556 | -5.785805556 |
| 40 | BLEKOUM | 6.36667 | -3.53306 |
| 41 | BLEMPLO | 8.08306 | -5.4 |
| 42 | BOKOBA | 8.86667 | -7.23306 |
| 43 | BOUNTOU | 8.49306 | -7.18056 |
| 44 | BOUROU M'PO | 6.211083333 | -4.246305556 |
| 45 | BOUROUNON | 7.73306 | -6.11667 |
| 46 | BOUSSANGASSO | 7.2 | -5.93306 |
| 47 | Boutoubré 2 | 5.841583333 | -6.740861111 |
| 48 | BRAHIMADOUGOU | 8.135861111 | -5.986861111 |
| 49 | BROUBROU | 5.722777778 | -4.823472222 |
| 50 | C.P.T MARAHOUE | 8.09972 | -6.26667 |
| 51 | CAMP. SOULEMANEDOUGO | 8.25 | -4.83306 |
| 52 | CAMPEMENT BARNABE | 6.225 | -3.62222 |
| 53 | Clement Bambakro | 6.958333333 | -5.499805556 |
| 54 | COHOTONKAHA | 8.924444444 | -6.421944444 |
| 55 | CPT SAHOUA | 6.337416667 | -5.171111111 |
| 56 | DABOKITILA | 7.61667 | -3.66667 |
| 57 | DABOYAOKRO | 7.55 | -3.5 |
| 58 | DANANGORO | 7.18306 | -7.93306 |
| 59 | DANTOGO | 7.81667 | -6.21667 |
| 60 | DEBETE | 10.6 | -6.65 |
| 61 | DIEOULIZRA | 7.48306 | -6.01667 |
| 62 | DIEOULIZRA 2 | 7.466055556 | -6.017222222 |
| 63 | DIKODOUGOU | 8.38306 | -5.76667 |
| 64 | DJIRILA-BADA | 10.10556 | -7.60167 |
| 65 | DOUMBA | 8.97139 | -7.33194 |
| 66 | DUPUY-YAO | 5.89944 | -4.8 |
| 67 | FANATOKODA | 8.33306 | -5.58306 |
| 68 | FARANDOUGOU | 9.21667 | -6.66667 |
| 69 | FD LOT (FEED LOT) | 8.35 | -6.36667 |
| 70 | FETEASSOU(BAKARIKRO) | 6.61389 | -4.73889 |
| 71 | FETEKRO | 7.81667 | -4.68306 |
| 72 | FLAMEBO | 9.45 | -5.35 |
| 73 | FODIOLOKAHA | 9.45 | -5.35 |
| 74 | FODONKAHA | 9.38333 | -5.28333 |
| 75 | GALEGOUA | 6.23306 | -4.83306 |
| 76 | GANSE | 8.62222 | -3.91472 |
| 77 | GBAGBA-SIRAKORO | 8.08306 | -4 |
| 78 | GBAHANLA | 9.529583333 | -8.120472222 |
| 79 | GBAMBIASSO | 9.76889 | -6.37222 |
| 80 | GBANANDOU | 8.377861111 | -7.704555556 |
| 81 | GBANDO | 9.56667 | -6.66667 |
| 82 | Gbanhala | 9.529583333 | -8.120472222 |
| 83 | GBOLY CARREFOUR(CAMP | 8.16667 | -4.61667 |
| 84 | GBORINAKAHA (TOUKAHA | 8.57833 | -5.89194 |
| 85 | GHANASSO | 8.36667 | -5.53306 |
| 86 | GLOLE | 7.23306 | -7.5 |
| 87 | GLOLEU I | 7.36667 | -7.75 |
| 88 | GNANGBATA | 9.57399 | -4.09094 |
| 89 | GNENINFOLOKAHA | 8.56667 | -5.61667 |
| 90 | GNONDJE | 8.85 | -6.48306 |
| 91 | GOLI N'ZIKRO | 7.339833333 | -4.360666667 |
| 92 | GOLI-N'ZIKRO | 7.34167 | -4.36583 |
| 93 | GOUE-GOUINE | 7.16667 | -7.68306 |
| 94 | GOUELE | 7.15 | -7.61667 |
| 95 | GUESSO-BONDOSSO | 8.28306 | -5.66667 |
| 96 | GUIENDANA | 9.21167 | -4.87917 |
| 97 | GUINGUERENI | 9.54111 | -6.58586 |
| 98 | Guinguéréni | 9.541111111 | -6.608611111 |
| 99 | JONASKRO | 7.06667 | -7.08306 |
| 100 | KADIOLA | 9.65528 | -7.62056 |
| 101 | KAFOLO | 9.585 | -4.31056 |
| 102 | KAHEN | 6.9 | -7.58306 |
| 103 | KAKOLOGO | 10.15 | -6.23306 |
| 104 | KAMALA | 8.45 | -2.68306 |
| 105 | KANGAKRO (5330) | 7.4 | -3.46667 |
| 106 | kassiolo | 10.45083333 | -6.520833333 |
| 107 | KAVAKA | 7.60194 | -6.09528 |
| 108 | KEBEKO | 9.6 | -6.9 |
| 109 | KEMISSIGA | 10.28333 | -7.53139 |
| 110 | KENANKITAKAHA | 9.039916667 | -6.694777778 |
| 111 | KENIGOUARA | 10.16111 | -7.78778 |
| 112 | KLAZRA | 7.45333 | -6.1125 |
| 113 | Kobadara | 9.203888889 | -4.981944444 |
| 114 | KOBADARA (POINT VII) | 9.20361 | -4.98194 |
| 115 | KOBALA | 9.01667 | -7.31667 |
| 116 | KODABAVOGO | 9.76444 | -5.70528 |
| 117 | Kodabavogo | 9.675277778 | -5.659166667 |
| 118 | KOFFIKRO | 6.906305556 | -5.670194444 |
| 119 | KOGOUEN | 7.38306 | -7.5 |
| 120 | KOHOLA | 8.60611 | -4.92806 |
| 121 | KOKOLOBA | 8.60611 | -4.92806 |
| 122 | KOKONOU | 7.13056 | -3.61806 |
| 123 | KOLOGNERIVOGO | 8.34525 | -6.22125 |
| 124 | KOLOKAHA | 8.94333 | -3.18167 |
| 125 | KOLOKAHA | 8.9 | -5.03306 |
| 126 | KONGOLO | 9.11778 | -4.49056 |
| 127 | KONGOLO | 9.11778 | -4.49056 |
| 128 | Kopkingué | 9.820833333 | -3.346666667 |
| 129 | KORODJALA | 9.09972 | -4.75778 |
| 130 | KOSSABA | 8.38361 | -3.99833 |
| 131 | Kossaba | 8.641222222 | -4.652213889 |
| 132 | KOTOBO | 7.73306 | -3.81667 |
| 133 | KOUADIOKRO | 7.0917 | -3.63145 |
| 134 | KOUAKOU KREMEKRO | 7.989972222 | -5.291694444 |
| 135 | KOUEZRA | 6.11667 | -6.18306 |
| 136 | KOULENLE | 7.15 | -7.51667 |
| 137 | KOUROUKORO | 7.95 | -6.41667 |
| 138 | KOUROUKOUNA | 9.28305 | -5.2 |
| 139 | KOUTOUKRO I | 5.61056 | -3.56583 |
| 140 | KOUTOUKRO II | 5.54917 | -3.53583 |
| 141 | KPOH 2 | 8.21375 | -8.1435 |
| 142 | KRAKRO | 7.53722 | -7.05667 |
| 143 | KROKOKRO | 6.63306 | -4.76667 |
| 144 | LAGBO | 9.71361 | -3.12444 |
| 145 | LAMEKAHA | 9.35 | -5.04972 |
| 146 | LAMOUEDOUGOU | 8.79917 | -7.849833333 |
| 147 | LAOUREBO | 6.5 | -4.78306 |
| 148 | LELE | 10.37667 | -7.63889 |
| 149 | LERABA-GARE | 10.11667 | -5.15306 |
| 150 | LEWARA | 8.8 | -4.35 |
| 151 | LIGBORO | 8.13306 | -4 |
| 152 | LISSOLO | 8.06667 | -4 |
| 153 | LOHO | 8.67917 | -5.13056 |
| 154 | LOMO NORD | 6.65 | -4.98306 |
| 155 | LONGO | 8.96667 | -5.4 |
| 156 | LONIENE | 8.31667 | -4.75 |
| 157 | MADINA | 10.28778 | -7.67667 |
| 158 | MAFA MAFOU | 5.847805556 | -4.069333333 |
| 159 | MAMORODOUGOU | 8.71667 | -7.33306 |
| 160 | MAMOUYA | 8.88306 | -7.53306 |
| 161 | MASSABOUEDOUGOU | 9.10833 | -7.375 |
| 162 | MASSADOUGOU | 9.05 | -7.41667 |
| 163 | MAZELA | 10.1925 | -7.54611 |
| 164 | MISSAMAHANA | 10.25 | -7.35 |
| 165 | Missasso | 10.6275 | -6.689444444 |
| 166 | MONEKOI | 5.54083 | -3.68028 |
| 167 | NABEDJAKAHA | 8.78306 | -5.53306 |
| 168 | NAFADOUGOU | 9.94445 | -7.56111 |
| 169 | NAGOUNGOKAHA | 8.70639 | -5.69028 |
| 170 | Nagoungokaha | 8.710444444 | -5.69525 |
| 171 | NAKELE | 9.86647 | -3.39225 |
| 172 | NALOURGOKAHA | 9.32167 | -5.70389 |
| 173 | NAMBAYEREDOUGOU | 8.71667 | -4.7 |
| 174 | Nambayérédougou | 8.726027778 | -4.892861111 |
| 175 | NAMILOHOKAHA | 8.45742 | -5.0735 |
| 176 | NANAKRI | 9.03667 | -5.49806 |
| 177 | NANIEVOGO | 9.78389 | -5.1425 |
| 178 | NAO | 8.25 | -2.88306 |
| 179 | NAWOLAVOGO | 9.71694 | -5.50028 |
| 180 | N'GAPIE | 9.56667 | -6.81667 |
| 181 | N'GORONDOUGOU | 8.38806 | -7.61917 |
| 182 | NIAMINAN | 10.07306 | -7.68167 |
| 183 | NIANDA | 5.53306 | -3.65 |
| 184 | NIANGORO | 8.01667 | -7.08306 |
| 185 | NIENESSO | 9.21667 | -7.4 |
| 186 | NIGUEDOUGOU | 9.162388889 | -6.720111111 |
| 187 | NOKOKRO | 7.53306 | -3.58306 |
| 188 | N'WODIOKRO | 6.01425 | -4.923666667 |
| 189 | NYANZONGO | 9.25194 | -7.06875 |
| 190 | OFFA GORKE | 5.92275 | -4.343777778 |
| 191 | OFFORIGUIE | 5.881111111 | -4.261666667 |
| 192 | OUATTARADOUGOU | 8.66667 | -5.81667 |
| 193 | OUEREGUEKAHA | 8.46667 | -5.3 |
| 194 | PANGALAKAHA | 9.08333 | -5.46667 |
| 195 | PAPARA | 10.63306 | -6.26667 |
| 196 | PEDEOUA | 7.75 | -3.85 |
| 197 | PEDERIKAHA | 9.41722 | -5.46833 |
| 198 | PEGON'SIONKAHA | 9.71194 | -5.08389 |
| 199 | PETIT NASSIAN | 9.3975 | -4.48028 |
| 200 | PETIT-YAPLEU | 7.10278 | -7.65278 |
| 201 | PIEBLY-DIOULABOUGOU | 7.28917 | -7.16306 |
| 202 | PIEYE | 8.88306 | -2.7 |
| 203 | PINGNEREKAHA (NAPIE) | 9.11667 | -5.41667 |
| 204 | POHIZRA I | 7.45 | -6.06667 |
| 205 | POSSEO | 9.11667 | -5.41667 |
| 206 | Poto-Poto | 6.913638889 | -5.591111111 |
| 207 | SAGOURA-SANON | 8.55 | -7.66667 |
| 208 | Salankourani | 9.723027778 | -7.804444444 |
| 209 | SALONKOURANI | 9.6 | -7.66667 |
| 210 | SAMANZA | 7.5 | -3.56667 |
| 211 | SAMATIGUILA | 8.81667 | -5.88306 |
| 212 | SAMOROSSOBA | 9.86917 | -6.3825 |
| 213 | SANGABILI | 8.36456 | -2.73067 |
| 214 | SAWA | 6.31667 | -5.16667 |
| 215 | SEFESSO | 9.11667 | -5.41667 |
| 216 | SEGUEBE | 9.17806 | -6.03528 |
| 217 | SENDRE SOKOURA | 8.73994 | -4.79486 |
| 218 | SETOUMOU | 8.9 | -6.66667 |
| 219 | SIRANA | 9.415861111 | -7.843777778 |
| 220 | SISSENGUE | 10.43306 | -6.21667 |
| 221 | SOKOURANE | 9.75695 | -7.58889 |
| 222 | SORIBADOUDOU | 8 | -7.06667 |
| 223 | SOROTANA | 8.08306 | -7.11667 |
| 224 | SOROTONA | 8.08306 | -7.11667 |
| 225 | SYENKOUNON | 9.03306 | -6.46667 |
| 226 | TAGADI (5129) | 8.63306 | -2.6 |
| 227 | TAKRA MANGOUAKRO | 7.842138889 | -5.389861111 |
| 228 | TANWARA-DJERO | 9.65083 | -5.65111 |
| 229 | TCHEBROU | 8.35 | -5.55 |
| 230 | TCHEDJESSO BADA | 8.460444444 | -6.73225 |
| 231 | TCHEDJOSSO | 8.45 | -6.73306 |
| 232 | TENIAKRO | 6.95 | -4.46667 |
| 233 | TIAPLEU | 7.2 | -7.71667 |
| 234 | TIEBEKAHA | 8.91667 | -5.58333 |
| 235 | TIEKORONIDOUGOU | 8.66667 | -7.36667 |
| 236 | TIEMBA | 8.13306 | -6.83306 |
| 237 | TIMANA | 8.13306 | -6.83306 |
| 238 | TOFESSO | 7.76667 | -6.06667 |
| 239 | TOGBASSO | 7.990722222 | -6.059972222 |
| 240 | TORO KINKENE II | 8.85222 | -4.42444 |
| 241 | TOUMBO | 7.83306 | -3.88306 |
| 242 | TOUMBO-YAGA | 8.53333 | -3.45 |
| 243 | TOURESSO 2 | 8.578 | -7.658 |
| 244 | TOURESSO II | 8.53333 | -7.66667 |
| 245 | TRIPOUNGO | 9.3125 | -5.75 |
| 246 | TRYPANO | 9.60181 | -3.68533 |
| 247 | Trypano | 9.771111111 | -3.8725 |
| 248 | TUOBA | 7.86667 | -7.46667 |
| 249 | TYEIFLA | 7.53272 | -6.05406 |
| 250 | TYONLE | 7.06667 | -7.63306 |
| 251 | VAKAVADOUGOU | 8.76167 | -7.7125 |
| 252 | VASSOFLA | 7.2 | -5.93306 |
| 253 | VIALADOUGOU | 8.54028 | -7.21861 |
| 254 | VIALADOUGOU | 8.54028 | -7.21861 |
| 255 | VONKORO MALI | 9.16744 | -2.73904 |
| 256 | VREHENFLA | 7.35 | -6.11667 |
| 257 | WAYARGAKAHA | 8.816222222 | -6.413305556 |
| 258 | WEBASSO | 8.25 | -7.16667 |
| 259 | WOROSSANTIAKAHA | 9.70722 | -5.09 |
| 260 | Worossantiakaha | 9.793333333 | -5.090777778 |
| 261 | YAMAUEKRO | 7.88306 | -4.63306 |
| 262 | YAOUDE | 6.56667 | -7.45 |
| 263 | YEALEU | 7.527361111 | -8.422027778 |
| 264 | YEDANDIEKAHA | 9.328 | -4.94475 |
| 265 | YEKOLO | 8.18306 | -4.91667 |
| 266 | YEREYERE | 6.23306 | -3.45 |
| 267 | YOMANKRO | 7.85 | -6.28306 |
| 268 | Zaébré | 5.317138889 | -6.275527778 |
| 269 | ZANAKAHA | 8.71667 | -5.55 |
| 270 | ZANASSO II | 9.91667 | -6.71667 |
| 271 | ZANOUKORO | 7.55 | -4.31667 |
| 272 | ZIDOKOUAHI | 7.235222222 | -6.103972222 |
| 273 | ZOBA | 7.28306 | -7.71667 |
| 274 | ZOGOUALE | 7.41667 | -7.53306 |
| 275 | ZONOKOI | 6.43306 | -3.61667 |
| 276 | ZOUZOUSSO II | 7.82083 | -7.65 |
