# Supplementary material for: Update on the current status of onchocerciasis in Côte d’Ivoire following 40 years of intervention: Progress and challenges
Source: PLoS Negl Trop Dis. 2018 Oct 23;12(10):e0006897. doi: 10.1371/journal.pntd.0006897 (PMC6214569; doi:10.1371/journal.pntd.0006897)
Supplement: S3 Table — (DOC) [file pntd.0006897.s004.doc]

Supporting information

S3 Table. Results of annual MDA rounds carried out continuously for onchocerciasis elimination in Cote d’Ivoire from 2008 to 2015

| **District** | **2008** | | | **2009** | | | **2010** | | | **2011** | | |
| --- | --- | --- | --- | --- | --- | --- | --- | --- | --- | --- | --- | --- |
|  | **Population** | **Treatment** | | **Population** | **Treatment** | | **Population** | **Treatment** | | **Population** | **Treatment** | |
|  | Eligible | No. treated | Therapeutic Coverage | Eligible | No. treated | Therapeutic Coverage | Eligible | No. treated | Therapeutic Coverage | Eligible | No. treated | Therapeutic Coverage |
| **Abengourou** | 46253 | 32333 | 70 | 39123 | 32037 | 82 | 44016 | 33950 | 77 | 41452 | 32456 | 78 |
| **Aboisso** | NT | NT | NT | 131392 | 95199 | 72 | 117904 | 85625 | 73 | 108836 | 83393 | 77 |
| **Adiake** | NT | NT | NT | 24859 | 17684 | 71 | 10659 | 8870 | 83 | NT | NT | NT |
| **Adzope** | NT | NT | NT | 2740 | 2428 | 89 | NT | NT | NT | 3299 | 2657 | 81 |
| **Agnibilekro** | NT | NT | NT | 10757 | 9006 | 84 | 18023 | 12114 | 67 | 15541 | 11859 | 76 |
| **Akoupe** | NT | NT | NT | 14468 | 10726 | 74 | 16100 | 10201 | 63.4 | 21070 | 16990 | 81 |
| **Alepe** | NT | NT | NT | 23273 | 14600 | 63 | 24639 | 16910 | 68.6 | 29100 | 22432 | 77 |
| **Beoumi** | 8547 | 6864 | 80 | 9727 | 8129 | 84 | 10340 | 8,708 | 84 | 8956 | 7473 | 83 |
| **Biankouma** | 13009 | 7507 | 58 | 18650 | 12025 | 64 | 18518 | 14542 | 79 | 23121 | 18595 | 80 |
| **Blolequin** | NT | NT | NT | 17584 | 11940 | 68 | 17895 | 14906 | 83 | NT | NT | NT |
| **Bocanda** | 55502 | 44181 | 80 | 59164 | 45777 | 77 | 62875 | 48525 | 77.2 | 58020 | 48023 | 83 |
| **Bondoukou** | NT | NT | NT | 49047 | 34197 | 70 | 60466 | 42068 | 73.3 | 54360 | 42548 | 78 |
| **Bongouanou** | NT | NT | NT | 26783 | 21828 | 82 | 28679 | 23126 | 80.6 | 23895 | 19998 | 84 |
| **Bouafle** | NT | NT | NT | 2772 | 2019 | 73 | 3318 | 2801 | 84.4 | 22523 | 18189 | 81 |
| **Bouake** | 13041 | 10070 | 77 | 15019 | 11118 | 74 | 16549 | 11341 | 69 | 16493 | 13125 | 80 |
| **Bouna** | NT | NT | NT | 10286 | 7872 | 77 | 11300 | 8288 | 73 | 13588 | 10814 | 80 |
| **Boundiali** | 3169 | 2256 | 71 | 2942 | 2619 | 89 | 3559 | 2348 | 66 | 3,646 | 1887 | 52 |
| **Dabakala** | 13413 | 10379 | 77 | 11133 | 8812 | 79 | 13934 | 11262 | 81 | 13,904 | 11717 | 84 |
| **Daloa** | NT | NT | NT | 31031 | 23235 | 75 | 34419 | 27196 | 79.1 | 41,281 | 33700 | 82 |
| **Danane** | 32376 | 23371 | 72 | 68642 | 50246 | 73 | 60112 | 44473 | 74 | 93,984 | 66277 | 71 |
| **Daoukro** | NT | NT | NT | 29142 | 20519 | 70 | 31686 | 24112 | 76.1 | 33,670 | 24654 | 73 |
| **Overall** | 185310 | 136961 | 73.1 | 598534 | 442016 | 75.5 | 604991 | 451366 | 75.6 | 626739 | 486787 | 77.9 |

NT: Not treated

| **District** | **2008** | | | **2009** | | | **2010** | | | **2011** | | |
| --- | --- | --- | --- | --- | --- | --- | --- | --- | --- | --- | --- | --- |
|  | **Population** | **Treatment** | | **Population** | **Treatment** | | **Population** | **Treatment** | | **Population** | **Treatment** | |
|  | Eligible | No. treated | Therapeutic Coverage | Eligible | No. treated | Therapeutic Coverage | Eligible | No. treated | Therapeutic Coverage | Eligible | No. treated | Therapeutic Coverage |
| **Dimbokro** | 35570 | 25021 | 70 | 37097 | 29988 | 81 | 36972 | 30765 | 83.2 | 35711 | 29644 | 83 |
| **Divo** | NT | NT | NT | 117597 | 74161 | 63 | 144286 | 112409 | 78 | 175766 | 133940 | 81 |
| **Grand Bassam** | NT | NT | NT | 8038 | 5414 | 67 | 6665 | 4899 | 74 | 10700 | 6840 | 64 |
| **Guiglo** | NT | NT | NT | 10852 | 6872 | 63 | 6623 | 4317 | 65.2 | NT | NT | NT |
| **Katiola** | 17900 | 12230 | 68 | 21628 | 13469 | 62 | 22073 | 17088 | 77 | 26652 | 21024 | 79 |
| **Korhogo** | 24547 | 17436 | 71 | 26233 | 21517 | 82 | 20916 | 13414 | 64 | 25929 | 21096 | 81 |
| **Lakota** | NT | NT | NT | 5741 | 4624 | 81 | 70940 | 53321 | 75 | 29613 | 20513 | 69 |
| **Man** | 55158 | 41524 | 75 | 70038 | 51168 | 73 | 77287 | 59185 | 77 | 103499 | 75908 | 73 |
| **Mankono** | 11400 | 7155 | 63 | 23812 | 18027 | 76 | 18203 | 9346 | 51 | 25111 | 20336 | 81 |
| **M’Bahiakro** | 15743 | 13179 | 84 | 19777 | 15077 | 76 | 19847 | 15543 | 78.3 | 27564 | 20094 | 73 |
| **Odienne** | 23428 | 16926 | 72 | 30225 | 22249 | 74 | 30298 | 22399 | 73.9 | 30070 | 23818 | 79 |
| **Oume** | NT | NT | NT | 13755 | 12053 | 88 | 17956 | 14250 | 79.4 | 17876 | 12572 | 70 |
| **Seguela** | 24024 | 18260 | 76 | 43555 | 33569 | 77 | 22746 | 17701 | 78 | 69759 | 52347 | 75 |
| **Tiassale** | NT | NT | NT | 61252 | 50372 | 82 | 44947 | 35938 | 79.9 | 122754 | 95272 | 78 |
| **Tiebissou** | NT | NT | NT | 14071 | 12023 | 85 | 18480 | 14800 | 80 | 21133 | 16826 | 80 |
| **Touba** | 28740 | 21490 | 75 | 38509 | 29697 | 77 | 54952 | 42826 | 78.8 | 70903 | 56072 | 79 |
| **Toulepleu** | NT | NT | NT | 20284 | 16014 | 79 | NT | NT | NT | NT | NT | NT |
| **Toumodi** | 28515 | 18716 | 66 | 42626 | 28785 | 68 | 44793 | 28672 | 64 | 61201 | 46006 | 75 |
| **Vavoua** | NT | NT | NT | 4006 | 3018 | 75 | 5090 | 3635 | 71.4 | 36768 | 26985 | 73 |
| **Yamoussokro** | NT | NT | NT | 17806 | 11679 | 66 | 24456 | 16013 | 65 | 22732 | 17825 | 78 |
| **Zuenoula** | NT | NT | NT | 46844 | 36018 | 77 | 57296 | 46146 | 81 | 62858 | 49790 | 79 |
| **Overall** | 265025 | 191937 | 72.0 | 605090 | 445079 | 74.8 | 657984 | 496873 | 73.7 | 854241 | 652308 | 76.3 |

NT: Not treated

| **District** | **2012** | | | **2013** | | | **2014** | | | **2015** | | |
| --- | --- | --- | --- | --- | --- | --- | --- | --- | --- | --- | --- | --- |
|  | **Population** | **Treatment** | | **Population** | **Treatment** | | **Population** | **Treatment** | | **Population** | **Treatment** | |
|  | Eligible | No. treated | Therapeutic Coverage | Eligible | No. treated | Therapeutic Coverage | Eligible | No. treated | Therapeutic Coverage | Eligible | No. treated | Therapeutic Coverage |
| **Abengourou** | 35620 | 28772 | 81 | 34484 | 28598 | 83 | 309430 | 25944 | 65 | 317475 | 259695 | 82 |
| **Aboisso** | 107987 | 79752 | 74 | 120901 | 97568 | 81 | 282489 | 257012 | 73 | 289834 | 241752 | 83 |
| **Adiake** | 19189 | 15433 | 80 | 17488 | 14921 | 85 | 127783 | 109645 | 69 | 131106 | 108852 | 83 |
| **Adzope** | NT | NT | NT | NT | NT | NT | 228217 | 190090 | 67 | 209136 | 154099 | 74 |
| **Agnibilekro** | 17508 | 14065 | 80 | 20334 | 16620 | 82 | 135524 | 111992 | 66 | 139048 | 115471 | 83 |
| **Akoupe** | 27203 | 22663 | 83 | 29833 | 25119 | 84 | 153906 | 145645 | 85 | 182058 | 148825 | 82 |
| **Alepe** | NT | NT | NT | 35606 | 27288 | 77 | 122407 | 106270 | 69 | 125590 | 108060 | 86 |
| **Beoumi** | 10195 | 8712 | 85 | 11582 | 9622 | 83 | NT | NT | NT | NT | NT | NT |
| **Biankouma** | NT | NT | NT | NT | NT | NT | 27431 | 21337 | 78 | 26883 | 21474 | 80 |
| **Blolequin** | NT | NT | NT | NT | NT | NT | 119276 | 112463 | 75 | 122378 | 102245 | 84 |
| **Bocanda** | 65483 | 52800 | 81 | 75408 | 61875 | 82 | 73137 | 60247 | 82 | 81652 | 68456 | 84 |
| **Bondoukou** | NT | NT | NT | NT | NT | NT | 373275 | 329004 | 71 | 382981 | 334671 | 87 |
| **Bongouanou** | NT | NT | NT | NT | NT | NT | 315034 | 274284 | 70 | 323225 | 266046 | 82 |
| **Bouafle** | 26791 | 20360 | 76 | 30013 | 23328 | 78 | 300629 | 267852 | 71 | 308446 | 261559 | 85 |
| **Bouake** | 19188 | 15588 | 81 | 19828 | 15569 | 79 | NT | NT | NT | NT | NT | NT |
| **Bouna** | NT | NT | NT | NT | NT | NT | 191442 | 165735 | 69 | 196419 | 175080 | 89 |
| **Boundiali** | NT | NT | NT | NT | NT | NT | NT | NT | NT | 3876 | 3075 | 79 |
| **Dabakala** | 18238 | 15305 | 84 | 18784 | 15562 | 83 | 131775 | 110275 | 67 | 135202 | 110126 | 81 |
| **Daloa** | NT | NT | NT | NT | NT | NT | NT | NT | NT | NT | NT | NT |
| **Danane** | NT | NT | NT | NT | NT | NT | 106998 | 84816 | 79 | 100007 | 80154 | 80 |
| **Daoukro** | 36013 | 27024 | 75 | 40503 | 31093 | 77 | 142722 | 115992 | 65 | 146434 | 119446 | 82 |
| **Overall** | 383415 | 300474 | 80.0 | 454764 | 367163 | 81.2 | 3,141,475 | 2,488,603 | 71.8 | 3,221,750 | 2,679,086 | 82.5 |

NT: Not treated

| **District** | **2012** | | | **2013** | | | **2014** | | | **2015** | | |
| --- | --- | --- | --- | --- | --- | --- | --- | --- | --- | --- | --- | --- |
|  | **Population** | **Treatment** | | **Population** | **Treatment** | | **Population** | **Treatment** | | **Population** | **Treatment** | |
|  | Eligible | No. treated | Therapeutic Coverage | Eligible | No. treated | Therapeutic Coverage | Eligible | No. treated | Therapeutic Coverage | Eligible | No. treated | Therapeutic Coverage |
| **Dimbokro** | 36387 | 28536 | 78 | 36593 | 29868 | 82 | 103,247 | 91,622 | 71 | 105932 | 86652 | 82 |
| **Divo** | NT | NT | NT | NT | NT | NT | NT | NT | NT | NT | NT | NT |
| **Grand Bassam** | 15158 | 12024 | 79 | 10170 | 8293 | 82 | 174,274 | 141,877 | 65 | 178805 | 151609 | 85 |
| **Guiglo** | NT | NT | NT | NT | NT | NT | 211,608 | 202,451 | 77 | 217110 | 177928 | 82 |
| **Katiola** | 12701 | 10027 | 79 | 12725 | 10646 | 84 | 95,549 | 87,446 | 73 | 98034 | 80642 | 82 |
| **Korhogo** | 39024 | 32872 | 84 | 36651 | 30713 | 84 | 36521 | 29,868 | 82 | 41290 | 34303 | 83 |
| **Lakota** | NT | NT | NT | NT | NT | NT | NT | NT | NT | NT | NT | NT |
| **Man** | NT | NT | NT | NT | NT | NT | NT | NT | NT | 89159 | 73269 | 82 |
| **Mankono** | 47062 | 34099 | 72 | 48580 | 36756 | 76 | 59374 | 47,044 | 79 | 58837 | 46495 | 79 |
| **M’Bahiakro** | 32673 | 26010 | 80 | 34593 | 27845 | 80 | 34478 | 27,979 | 81 | 37971 | 31225 | 82 |
| **Odienne** | 31240 | 23886 | 76 | 66909 | 51436 | 77 | 200,037 | 175,847 | 70 | 205238 | 168231 | 82 |
| **Oume** | 21344 | 16533 | 77 | 23352 | 18550 | 79 | NT | NT | NT | NT | NT | NT |
| **Seguela** | 61399 | 49133 | 80 | 72566 | 58562 | 81 | NT | NT | NT | NT | NT | NT |
| **Tiassale** | NT | NT | NT | NT | NT | NT | 223,964 | 182,234 | 65 | 229795 | 172140 | 75 |
| **Tiebissou** | 23292 | 17811 | 76 | 22988 | 17896 | 78 | 90,753 | 77,137 | 68 | 93112 | 66175 | 71 |
| **Touba** | 63485 | 47247 | 74 | 66210 | 53266 | 80 | 75823 | 62,027 | 82 | 79019 | 64516 | 82 |
| **Toulepleu** | NT | NT | NT | NT | NT | NT | 64,362 | 60,378 | 75 | 66035 | 55488 | 84 |
| **Toumodi** | 59476 | 45439 | 76 | 52977 | 42800 | 81 | 134,411 | 116,406 | 69 | 134592 | 126436 | 94 |
| **Vavoua** | NT | NT | NT | NT | NT | NT | NT | NT | NT | NT | NT | NT |
| **Yamoussokro** | 22836 | 17608 | 77 | 30453 | 24989 | 82 | NT | NT | NT | NT | NT | NT |
| **Zuenoula** | 70773 | 57049 | 81 | 72942 | 59900 | 82 | NT | NT | NT | NT | NT | NT |
| **Overall** | 443,241 | 343,617 | 77.8 | 484,314 | 386,631 | 80.6 | 1,504,401 | 1,302,316 | 73.6 | 1,634,929 | 1,335,109 | 81.8 |

NT: Not treated
